# Using the Aesop's Fable Paradigm to Investigate Causal Understanding of Water Displacement by New Caledonian Crows

**DOI:** 10.1371/journal.pone.0092895

**Published:** 2014-03-26

**Authors:** Sarah A. Jelbert, Alex H. Taylor, Lucy G. Cheke, Nicola S. Clayton, Russell D. Gray

**Affiliations:** 1 Department of Psychology, University of Auckland, Auckland, New Zealand; 2 Department of Psychology, University of Cambridge, Cambridge, United Kingdom; CNR, Italy

## Abstract

Understanding causal regularities in the world is a key feature of human cognition. However, the extent to which non-human animals are capable of causal understanding is not well understood. Here, we used the Aesop's fable paradigm – in which subjects drop stones into water to raise the water level and obtain an out of reach reward – to assess New Caledonian crows' causal understanding of water displacement. We found that crows preferentially dropped stones into a water-filled tube instead of a sand-filled tube; they dropped sinking objects rather than floating objects; solid objects rather than hollow objects, and they dropped objects into a tube with a high water level rather than a low one. However, they failed two more challenging tasks which required them to attend to the width of the tube, and to counter-intuitive causal cues in a U-shaped apparatus. Our results indicate that New Caledonian crows possess a sophisticated, but incomplete, understanding of the causal properties of displacement, rivalling that of 5–7 year old children.

## Introduction

As adult humans we are capable of recognising that objects in the world behave in predictable ways. For example, we know that two objects cannot occupy the same space, round objects will roll down hills, and heavy objects sink in water. Many of these expectations are present very early in life [1], [2], whilst others emerge and evolve over the course of development [3]. It is easy to imagine that an ability to attend to causal regularities in the world, and to understand the forces underlying them, would have adaptive significance for many animal species. Whether animals do attend to causal regularities has been studied using various methodologies in different species (for review see [4]). However, finding comparative tasks to assess how causal information is processed by different species can be difficult. Existing tasks are often tied to specific ecologically relevant behaviours such as tool use (e.g. [5], [6]), involve face-to-face interactions with humans [7], or are too cognitively challenging to be attempted by more than a select few animals [8].

The most widely used paradigm – the trap-tube task – has been employed to investigate whether causal understanding underlies the natural tool-use found in some species of primates [9]–[13] and birds [14], [15]. In this task, an animal uses a stick to push or pull a food reward out of a perspex tube, avoiding a visible hole in the centre of the tube where the food would become trapped. Whilst trap-tube results have been interpreted comparatively to indicate differences between the cognitive abilities of great apes and monkeys [16], and between humans and other animals [9], as well as similarities between chimpanzees and corvids [17], [18], the capacity of this paradigm to test causal understanding is undermined by several significant limitations [19]. First, even slight changes to the task alter performance within a species. Apes, which predominantly failed standard trap-tube experiments [9]–[11], were much more successful when they could pull rather than push food out of the tube [20], and all subjects passed when they were not required to use a tool at all [17]. Second, errors made by animals on a key transfer task, the inverted trap-tube – where the trap is presented in a non-functional position on the upper surface of the tube – were also made by adult humans [21], suggesting that errors on this task cannot confirm an absence of causal understanding. Third, although the trap-tube has now been made accessible for non-tool using animals [22], the initial task is still difficult for most animals to grasp. The majority of subjects tested either fail or take a long time to perform successfully (e.g. 2 out of 5 chimpanzees passed over 140 trials [10], 3 out of 6 New Caledonian crows passed over 150 trials [15], and 7 out of 8 rooks passed over 150 trials [18]), thus floor effects preclude its use with less capable species. With this in mind, it is difficult to argue that the failures of some animals on aspects of the trap-tube reflect an absence of causal understanding in that species.

The recently-devised Aesop's fable paradigm may be a more informative paradigm for testing causal understanding across a wide range of species. This paradigm was initially used to test physical cognition in rooks by Bird & Emery [23], and is based on Aesop's well-known tale ‘the Crow and the Pitcher’. In this story a thirsty crow drops stones into a half-full pitcher of water, raising the water level in the pitcher until it is high enough for the crow to drink. In the equivalent experiment subjects are presented with a pile of stones and a tube of water containing a floating reward, such as a worm or meat on a cork. Bird & Emery found that rooks who had experience of dropping stones [24], but not in the context of water, would spontaneously drop stones into this water-filled tube to raise the water level and obtain the reward. This task bears considerable similarity to the ‘floating peanut’ task, in which apes and children will spit or pour water into a container to obtain out-of-reach floating rewards [25], [26]. The strength of both these paradigms lies in their ability to examine the reaction of animals to novel problems that are not related to the animal's habitual or customary tool use behaviours [27].

In subsequent experiments Bird and Emery found that rooks would preferentially drop large stones than small stones, would drop stones into water rather than into a tube containing sawdust, and tended to match the number of stones they dropped to the level of the water, only reaching into the tube once the worm was within their grasp. Whilst it shouldn't be assumed that the rooks planned their actions in advance [28], this does indicate that the rooks' stone-dropping behaviour was goal-directed, with the intention of obtaining the worm, and that they either understood or had quickly learnt several causal features of the task; i.e. that objects must be dropped into a liquid, and that large objects are more functional than small objects. This paradigm can therefore be used as a test of causal cognition, investigating whether animals can understand or learn about various causal regularities which underlie the displacement of water.

Replications using the Aesop's fable paradigm with other species – New Caledonian crows (hereafter NC crows), Eurasian jays and human children – have confirmed that subjects are more able to use causal information than arbitrary information to obtain the reward in these tasks [29]–[31]. NC crows [31] quickly discriminated between large and small stones, heavy and light objects, and water-filled and sand-filled tubes in the causal stone dropping paradigm. However, they then failed a series of associative learning controls in which the previously-rewarded stimuli (large objects and water-filled tubes) now indicated the location of food in an arbitrary searching paradigm. Similarly, Cheke and colleagues [30] found that at least one Eurasian jay performed above chance on all of their causal water-displacement conditions. However, the jays were not as successful when given similarly rewarded conditions which followed arbitrary rules. No birds preferentially dropped stones into a red woodchip tube instead of a blue woodchip tube (or vice versa) when one of these tubes was rewarded, suggesting that instrumental conditioning alone cannot explain the pattern of results. Furthermore, the birds were less successful when the reward was moved incrementally towards the bird after each stone drop in a non-causal L-shaped apparatus. Thus, like NC crows, Eurasian jays used causal cues, in combination with instrumental learning, to solve the water-displacement tasks.

In a further experiment of the study by Cheke and colleagues, jays [30] (and subsequently children [29]) were presented with an apparatus that made use of counter-intuitive causal cues. Referred to as the ‘U-Tube’ apparatus, it consisted of a narrow central tube containing a floating reward, with wider, coloured, tubes positioned on either side. One of the wider tubes had a concealed connection to the central tube underneath the table, but the other did not. Therefore, only stones that were dropped into the wide outer tube with a concealed connection to the central tube would raise the water level in the rewarded central tube; adding stones to the other wide tube that was not connected to the central tube would not raise the water level. The causal relation in this task was counter-intuitive: putting a stone into one body of water would raise the water level in another body of water. Thus, if subjects' actions were guided largely by a basic understanding of causality (which could not account for a hidden causal mechanism) their performance should be selectively impaired on this task (see [30]). On the other hand, if subjects relied on an associative rule or simply made quick reactions to perceptual feedback – repeating certain actions that bring the food closer within reach [28], [32] – they would be able to succeed. Alternatively, subjects could also pass by using a robust understanding of displacement to posit the existence of a hidden causal mechanism, that one of the outer tubes is connected to the central tube, underneath the table, allowing the displaced water to move between both tubes. If subjects succeeded on this task, the strategy they used to succeed could be identified by verbal response (for children), or in follow-up studies, by revealing the hidden connection, or changing the colours of the outer tubes.

The majority of eight-year-old children, and some younger individuals, solved this task. While some older children inferred the presence of the hidden connection, most succeeded by identifying which outer tube was the ‘correct’ tube to drop stones into. Thus, the primary method children used to solve the task was an associative rule. At all ages, the children's success rate on the U-tube task was similar to the other tasks, which followed intuitive causal rules. In contrast, Eurasian jays selectively failed the U-tube, suggesting that they struggled to learn a rule that contradicted their understanding of how the world *should* work. Thus, the difficult U-tube task can be used to investigate different types of reasoning about displacement.

In the current study we used the Aesop's fable paradigm to examine causal understanding in NC crows, producing a series of tasks to tap the extent to which animals can understand or learn about the causal features of displacement. NC crows are a strong candidate for understanding this type of causal information. These birds have exceptional tool manufacturing abilities, routinely making and using tools in the wild [33]–[35] as well as in captivity [5], [36]. They attend to functional properties of their tools, such as length and diameter [37], [38], and demonstrate causal understanding in both tool-using [15], [39], [40] and non-tool using contexts [41]. Previous work using the Aesop's fable paradigm has hinted that NC crows may understand the causal features of displacement [31], but further tests are required to probe the extent of their understanding.

In a progressive series of tests we replicated past experimental conditions and then investigated whether these birds would choose solid instead of hollow objects, narrow rather than wide tubes, high rather than low water levels, and whether they would succeed or fail with the causally confusing U-tube task [29], [30]. If the birds fully understood the causal relations involved in displacement they should recognise that solid objects displace more water than hollow objects, and preferentially drop solid objects into the tubes. They also should recognise the effect that changes in magnitude have on displacement and understand that objects dropped into a narrow tube will raise the water level by a greater amount than objects dropped into a wide tube. They should be sensitive to the starting water levels of different tubes; whilst a narrow tube would typically be preferable, a wide tube would become the more efficient option if the starting water level in this tube was substantially higher than the narrow tube. Finally, a basic understanding of displacement might impair the crows' performance on the causally confusing U-tube apparatus (as seems to be the case for Eurasian jays), but rapid associative learning, or an ability to infer a hidden connection between the outer tube and rewarded central tube, would enable birds to succeed.

Searching for the signature hallmarks of cognitive mechanisms, in terms of both when and why animals fail aspects of a task, in addition to when they succeed, can provide a much richer understanding of cognition than examining successes alone [42]. Thus, each experiment in our series of tasks was designed to assess NC crows' causal understanding of displacement from a slightly different perspective. In doing this, we could inspect the pattern of successes and failures across all of the experiments, to gain an understanding of how these birds solve displacement tasks. To ensure the birds' experience could be comparable with previous experiments a standardised order of tasks was used for all birds. Six tasks were given in all: (1) water-filled tubes vs. sand-filled tubes, (2) sinking objects vs. floating objects, (3) solid objects vs. hollow objects, (4) narrow tubes vs. wide tubes, (5) high water level vs. low water level in the narrow and wide tubes, and (6) the counter-intuitive U-tube task.

## Methods

### Ethics Statement

This study was conducted under approval from the University of Auckland ethics committee (reference no. R602).

### Subjects

Subjects were six wild NC crows, caught and temporarily housed in a six-cage aviary on Grande-Terre, New Caledonia. Three of the birds (R, W & Y) were adults and three were sub-adults (O, RB & WG). Based on sexual size dimorphism [43] two of the birds (R & Y) were male. Two birds (WG & R) did not complete all the experiments due to lack of motivation; thus, six birds took part in Experiments 1 & 2, five birds took part in Experiments 3 & 4, and four birds took part in Experiments 5 & 6. Birds were released at their site of capture at the end of this experiment.

### Initial Training

NC crows drop candlenuts on to hard surfaces to break them [44]. However, they are not known for dropping stones on to objects in the wild, nor are they known to drop stones into water. Therefore, before the experiment proper began, the crows were trained to drop stones into a tube to collapse a platform using a replica of Bird & Emery's apparatus [24] (for diagram see Figure S1).

Birds initially used a stick and inserted it into the tube to collapse the platform. They were then trained to nudge stones down the tube, until they would pick up stones from the table to drop into the apparatus. Once birds had successfully dropped stones down the tube 10 times, a second apparatus was provided with a weighted platform which would collapse when multiple stones were dropped onto it (Figure S1). Birds were given experience with this apparatus until they obtained the reward using 2–4 stones on 10 consecutive trials.

### General Procedure

Following training birds took part in 6 experiments in which they dropped objects into water-filled tubes, raising the water level to obtain a floating out of reach reward. In all cases the reward was a cube of meat attached to a cork.

**Figure 1:**
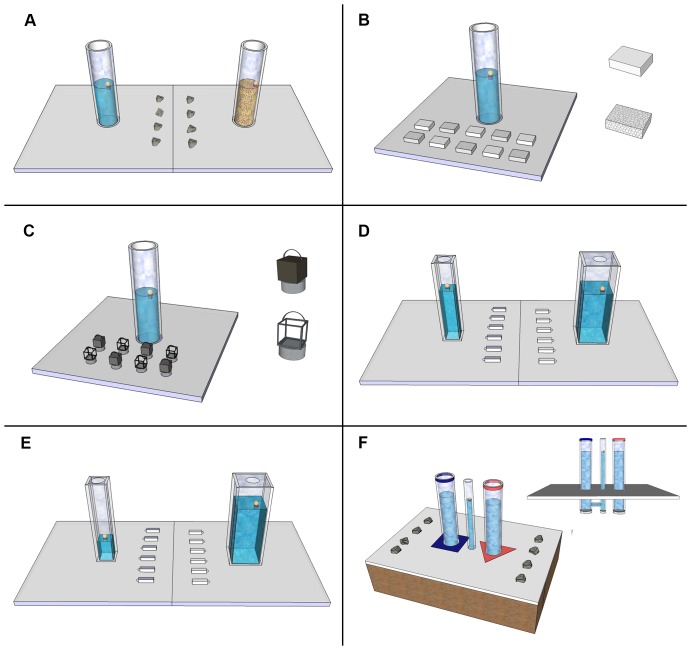
Diagrams of the apparatus used in each of the 6 experiments. In each experiment birds dropped objects into tubes to obtain an out of reach food reward. Each experiment involved either a choice of two tubes or a choice of two objects. The apparatus was presented on a table in the centre of a large testing cage, as pictured. A: Experiment 1, Sand-filled tubes v Water-filled tubes, B: Experiment 2, Sinking v Floating objects, C: Experiment 3, Solid v Hollow objects, D: Experiment 4, Narrow v Wide tubes, E: Experiment 5, High v Low water levels in Narrow and Wide tubes, F: Experiment 6, U-tube, a concealed connection links one of the outer tubes with the rewarded central tube.

First, we established how far each bird could reach into a water-filled tube with their beak, by presenting floating food rewards at different water levels. Then, birds were habituated to the task components. Birds were given a minimum of ten habituation trials to tubes (taking scraps of meat from the top and base of each tube), and three to objects (taking meat from under and on top of each object). Once habituated, birds were given 20 experimental trials on each of the six tasks, typically in two blocks of 10 trials. Tubes were pseudo-randomly presented on each side, no more than twice on one side, and the birds' choices of objects or tubes were recorded. Choices were defined as picking up an object from the table and dropping it into a tube. In some conditions it was possible for the bird to take inserted objects out of the tube, and place them back on the table, in which case the number of choices could be larger than the total number of objects. In experiments involving two spatially separated tubes (Exps. 1, 4 & 5) birds were given up to 30 seconds at the beginning of a trial to inspect both tubes before the stones or objects were placed on the table. Trials ended when the bird obtained the meat, used all of the objects, or twice left the table without interacting with the apparatus. Choices were analysed in terms of the group-level preferences and for each bird individually using binomial tests, with Bonferroni corrections to account for multiple tests. Birds were tested in visual isolation in a separate cage within the aviary. All trials were recorded on video. For diagrams of each experimental apparatus see Figure 1. For individual performance data see Data S1.

### Specific Procedure and Materials

#### Experiment 1: Water- vs. Sand-filled tubes

Two clear Perspex tubes (170 mm high, ID: 40 mm, OD: 50 mm), mounted onto 300×300 mm Perspex bases, were placed on the table, one half-filled with water and one half-filled with sand. The water and sand levels were set at 12 mm below each bird's reachable height. Eight stones of similar size, each weighing approximately 15 g, were arranged in between the two tubes. Each stone would displace 3–4 mm of water in the water-filled tube, but have no functional effect in the sand-filled tube.

#### Experiment 2: Sinking vs. Floating objects

A single water-filled tube (as used in Exp. 1) was placed on the table. Five heavy and five light objects of the same size and colour were arranged, in an alternating pattern, in front of the tube. Heavy objects were made from rubber (commercially available erasers), weighed 18 g and would sink to displace 6 mm of water in the tube. Non-functional light objects were made from polystyrene, weighed <1 g, and would float on the surface of the water.

#### Experiment 3: Solid vs. Hollow objects

A single water-filled tube was placed on the table with four solid and four hollow objects (arranged as in Exp. 2). Solid objects were made from of a cube of fimo modelling clay, with an empty metal cap attached to the base. Hollow objects were shaped from bended wire, attached to a metal cap containing a bolt and fimo clay to balance the weight. Thus both objects were similarly coloured, and were the same size and weight (15 g), however solid objects would displace 7 mm of water in the tube, whilst hollow objects would displace only 2 mm.

#### Experiment 4: Narrow vs. Wide water-filled tubes

Two differently-sized square tubes (both 170 mm high; attached to 300×300 mm Perspex bases) were placed on the table. The top surface of the narrow tube had an area of 36 mm^2^ (inner area: 24 mm^2^), and the wide tube had an area of 56 mm^2^ (inner area: 44 mm^2^). The volume of each tube was equally larger or smaller than the circular tube used in experiments 1–3. Both tubes were lidded, with a circular hole in the centre of the lid (D: 24 mm) through which objects could be dropped. Twelve objects – thin rubber blocks, 40×10×10 mm, weighing 9 g each – were arranged in between the two tubes. These blocks would displace 8 mm of water in the narrow tube, but only 2 mm of water in the wide tube. The narrow and wide tubes had slightly different reachable heights as the reward could float further to the side and out of reach in the wide tube (a difference in reachable height of 0–5 mm depending on reward position). To avoid giving subjects exposure to this, an additional bird, not otherwise involved in the experiment, was used to establish the appropriate water level. The equivalent level for each bird was estimated based on their reachable heights in the circular tube. The water level for both tubes was set at 12 mm below the reachable distance for the narrow tube.

#### Experiment 5: High vs. Low water-level in Wide & Narrow water-filled tubes

The materials were identical to those used in Exp. 4, except that the water levels in the two tubes were not equal. In the wide tube the water level was set at 6 mm below the reachable distance for the wide tube for each bird (i.e. ∼120 mm); in the narrow tube it was set consistently at 50 mm. With 50 mm of water it was impossible to raise the water enough to bring the food within reach, so the wide tube was the only functional choice.

#### Experiment 6: U-Tube

The U-tube apparatus was closely modelled on Cheke and colleagues apparatus used with Eurasian jays and children [29], [30]. It consisted of three tubes, positioned 25 mm apart in the centre of a 400×300 mm opaque Perspex base. The three tubes extended 170 mm above, and 70 mm below the base. The two outer tubes (OD 40 mm, ID 30 mm) were un-baited tubes into which stones could be dropped. The middle tube (OD 20 mm, ID 14 mm) was baited, but was too narrow for stones to be dropped into. One of the outer tubes was connected to the middle tube underneath the base, such that stones dropped into this tube would raise the water level in both this tube and the middle tube, bringing the reward within reach. The other tube was unconnected, so stones dropped into this tube would have no effect on the water level in the middle tube. As the mechanism was concealed, to discriminate between the connected and unconnected outer tubes one was marked with a blue rim and a blue square around the tube base, the other with a red rim and a red triangle. For three birds the red triangle marked the connected tube, and for one bird it marked the unconnected tube. Following habituation, the reachable height for the middle tube was established for each bird, and the water level for all three tubes was set at 12 mm below this level. Eight stones of a similar size, weighing 12 g, were presented, with 4 stones to the side of each tube.

## Results

Four of the six birds took part in all of the experiments. Two birds (WG & R) did not complete all experiments due to diminished motivation. These birds stopped after Experiment 4 and Experiment 2, respectively. Thus, six birds took part in Experiments 1 & 2, five birds took part in Experiments 3 & 4, and four birds took part in Experiments 5 & 6. See Movie S1 for an example trial from each experiment.

### Results from each Experiment

#### Experiment 1: Water- vs. Sand-filled tubes

**Figure 2:**
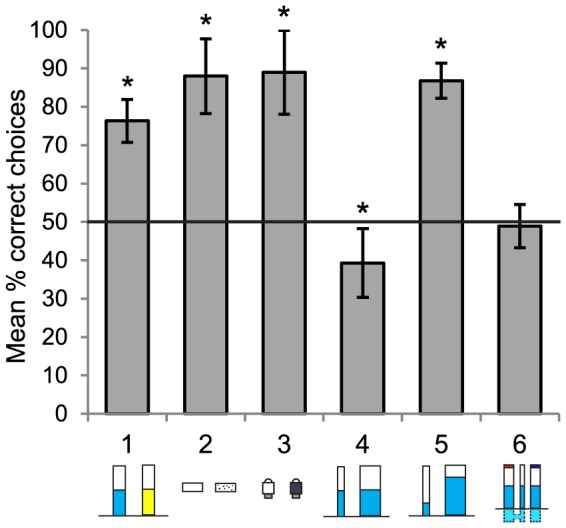
Average performance in all six experiments. Mean proportion of choices made to the correct option, over 20 trials, in each experiment. (1: Sand v Water, 2: Sinking v Floating, 3: Solid v Hollow, 4: Narrow v Wide, 5: High v Low water levels, 6: U-tube.) Error bars are ±2 SE. *  =  significantly different from chance (binomial tests, *p*<0.001).

All birds dropped significantly more stones into the water-filled tube (76.3% of stone drops), than the sand-filled tube, across 20 trials (binomial test, *p* = 0.001, Figure 2). Individually five out of six birds' performance reached significance within 15 trials (binomial test, *p* value <0.001), the remaining bird (R) approached significance at 20 trials (binomial test, *p* = 0.01, ns with a Bonferroni adjusted alpha level of 0.007).

#### Experiment 2: Sinking vs. Floating objects

All birds dropped sinking objects (88.0% of choices) into the water-filled tube more often than floating objects across 20 trials (binomial test, *p*<0.001). One bird (R) picked up and discarded the floating object 16 times, but never dropped a floating object into the tube. Across the experiment, birds discarded the floating objects 65% of the times they picked one of them up, and discarded the sinking object on 0.02% of pickups, which was significantly different (paired t-test, *t* (5) = 6.21, *p* = 0.002). Individually, all birds demonstrated a significant preference for the sinking object; four birds did so within 10 trials and two birds (O & WG) did so within 20 trials (binomial test, *p*<0.001).

#### Experiment 3: Solid vs. Hollow objects

All birds dropped solid objects (89.0% of choices) into the tube more often than hollow objects, across 20 trials (binomial test, *p*<0.001). Four of the five birds reached significant individual performance in eleven trials or less (binomial test, *p*<0.001), the remaining bird (W) reached significance by the 20th trial (binomial test, *p* = 0.005, significant with a Bonferroni adjusted alpha level of 0.008). No bird selected a hollow object on their first trial, and two of the five birds (RB & Y) never dropped a hollow object into the tube. There was no difference in the proportion of solid and hollow objects discarded (hollow: 28% of pickups, solid: 0.04% of pickups, paired t-test, *t* (4) = 1.75, *p* = 0.15).

#### Experiment 4: Narrow vs. Wide water-filled tubes

**Figure 3:**
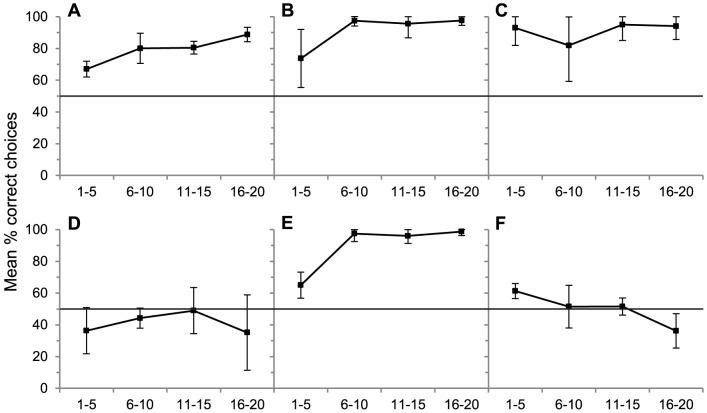
Changes in mean performance over the course of each experiment. Each panel gives the mean percent correct choices over trials 1–5, trials 6–10, 11–15 and 16–20, in each experiment, to indicate changes in performance over the course of 20 trials. (A: Sand v Water, B: Sinking v Floating, C: Solid v Hollow, D: Narrow v Wide, E: High v Low water level, F: U-tube.) Error bars are ±2 SE.

Birds did not drop more objects into the more efficient narrow tube (39.3% of object drops) than the wide tube, in fact in total they dropped significantly more objects into the wide tube (binomial test, *p*<0.001). However, the birds were able to obtain the reward from both tubes, and they frequently dropped objects only into the first tube they selected, rather than switching between the two tubes. On average birds retrieved the reward from the wide tube after 7 object drops, and from the narrow tube after only 2 drops. Thus, birds probably dropped more objects into the wide tube simply because, if they chose this tube first, more objects were required to obtain the reward. Importantly, on their first object drop per trial, across 20 trials, birds showed no preference for either the narrow or wide tube (narrow tube chosen first on 56% of trials, binomial test, *p* = 0.27), and individually no bird dropped significantly more objects into either tube. Birds showed no sign of developing a preference for the narrow tube over 20 trials (see Figure 3).

#### Experiment 5: High vs. Low water-level in Wide & Narrow water-filled tubes

Across 20 trials all birds dropped more objects into the wide tube with high water level (86.8% of choices) than the narrow tube with a non-functional low water level (binomial test, *p*<0.001). All birds dropped 3 or more objects into the narrow tube on their first trial, but then learnt to avoid this tube (indicated in Figure 3), with each bird reaching significance individually between trials 6–11 (binomial test, *p*<0.001).

#### Experiment 6: U-Tube

All birds performed at chance levels, dropping on average 48.9% of stones into the connected tube over 20 trials (binomial test, *p* = 0.79). They showed no signs of learning which tube would bring the reward within reach over the course of the experiment, performing worse in later trials due to increasing tendencies to side bias and repeatedly drop items into one tube rather than switching between both tubes (Figure 3). An inspection of each bird's individual stone drops (Data S1) did not reveal any patterns suggesting that birds responded to perceptual-motor feedback (i.e. repeat actions which bring the food closer, switch if the action does not).

## Discussion

Taken together, the results presented here show that the NC crows we tested were successful on some, but not all, of the displacement experiments. In line with previous work [31], they preferentially dropped stones into water-filled tubes rather than sand-filled tubes, and they dropped sinking objects more often than floating objects. This performance is comparable to 5- to 7-year old children, who learned to pass similar versions of these tasks over the course of 5 trials [29]. NC crows also attended to the water level of the tubes, dropping more objects into a tube with a high rather than low starting water level. Intriguingly, in the current study NC crows also demonstrated strong preferences to drop solid objects rather than hollow objects into the water-filled tubes. This is the first time an understanding of solidity has been studied in this paradigm, and the fact that NC crows are successful on this task supports the claim that they have a causal understanding of displacement. In all experiments in which they were successful, the birds demonstrated rapid learning.

Before they took part in the water-based experiments, all birds were trained to drop stones into a Perspex apparatus with a collapsible platform. Their ability to drop stones and other objects into the water-filled tubes in the experimental conditions is therefore not a result of insightful problem solving (unlike [45], for example). However, during training birds were provided with natural stones only, they did not have previous experience dropping light and heavy objects, or solid and hollow objects into the apparatus, and they did not have experience dropping stones into sand or water before the experiment began. The birds' specific preferences for the correct tubes and correct objects in these four experiments are therefore difficult to explain as the result of an associative rule learnt during training.

To be comparable with previous experiments [29]–[31] all birds received the tasks in a fixed order. Thus, it is possible that experience with objects and water on the earlier tasks facilitated the bird's performance on later tasks. Presenting the experiments in a different order could rule this out; however, given that a different physical property was relevant in each experiment (i.e. different substrates, weights, solidities, or volumes of water), prior experience is unlikely to fully account for success on the four different tasks.

In contrast to their success on four of the tasks, NC crows did not differentiate between narrow and wide tubes, nor did they succeed on the causally confusing U-tube task. As only a small number of birds took part in this study, we cannot assume this reflects a species-wide failure. It is notable, however, that all of the NC crows tested failed these two tasks, whereas all of the NC crows reached significance, or approached significance (one bird on one experiment) in the remaining four tasks, indicating that the U-tube and the narrow vs. wide tubes tasks are considerably harder for NC crows to pass.

In the narrow and wide tubes experiment, NC crows showed no preference for the narrow tube on their first object drop per trial, and overall dropped more objects into the wide tube, rather than the narrow tube, to obtain the reward. Individually, no bird had a preference for either tube. It is possible that this lack of success was because they could not distinguish visually between the two tubes. However, this seems unlikely as the wide tube was more than double the size of the narrow tube, and NC crows have excellent vision [46]. Alternatively, since the birds could eventually retrieve the reward from either tube, the difference was only one of effort, and this may have provided insufficient motivation to prefer the narrow tube. This also seems unlikely in the light of previous work showing that the NC crows preferred to drop efficient large stones rather than small stones into tubes [31] and the observation that some of the crows began to lose motivation towards the end of this task. If these birds understood the quickest way to access the food, it is likely that they would have applied this strategy on every trial.

It is intriguing then, that birds passed the solid and hollow objects condition but failed the narrow and wide tubes, given that the amount of water displaced was equivalent in both. This result could have stemmed from differences in the size, proximity or quantity of the objects and tubes, or it could reflect the difficulty of recognising that the volume of a tube can be a relevant causal property, compared to the potentially more salient properties of solidity or weight. It could also reveal a more general principle: it may be easier for this tool-using species to recognise the functional properties of various *tools* than to recognise the functional properties of the substrates their tools interact with. In support of this, although birds passed the sand- vs. water-filled tubes condition, they made more errors on this task than on either of the object discrimination tasks. Confirming reasons for the birds' failure requires further research, but our results do suggest a limit on NC crows' understanding of displacement, at least in this sample. These birds potentially possess a heuristic that ‘objects dropped into water make the water-level rise’, and can discriminate amongst the different causal properties of these objects; however, the relationship between tube width and magnitude of water displacement does not seem to be something these crows are aware of, or something that they can quickly learn.

The final experiment tested whether NC crows would pass or fail the causally confusing U-tube task. Success on this task could be achieved in two distinct ways: (1) at a low level, by relying on perceptual-motor feedback to repeat actions which bring the reward incrementally closer[28], [32], or by associating one of the coloured outer tubes with reward, or (2) at a high level, by inferring a hidden connection between two of the tubes, which would allow water to pass between them. Children passed this task reliably from 8 years of age, and individually 4- to 10-year-old children reported using both of these strategies, but only the low level rule was statistically related to success [29]. However, Eurasian jays failed this task [30] despite passing other causally-consistent tasks, suggesting that a basic understanding of causality – of how things *should* work – prevented them from using any other information to succeed. The results from the current U-tube experiment suggest that NC crows are comparable to Eurasian jays, but differ from human children. NC crows performed at chance, showing no signs that they could learn the identity of the correct tube over 20 trials. Thus, they were clearly unable to use a full understanding of displacement to infer the existence of a hidden connection, nor could they solve the task using only perceptual feedback or associative rules. This suggests the possibility that, like Eurasian jays, the NC crows possess a level of causal understanding which hindered their performance on this task with counter-intuitive causal cues.

It is perhaps surprising that birds could not use perceptual-motor feedback alone to solve the U-tube. NC crows are known to depend on perceptual-motor feedback to spontaneously solve string pulling tasks [32], [47], based on findings that the performance of both experienced and naïve birds is significantly impaired when visual access to the string is restricted. However, in an alternative string-pulling paradigm [48] inexperienced ravens could not solve a counter-intuitive task (where the string had to be pulled down, to move the reward up), despite the solution involving a comparable action pattern to a standard string pulling task. Thus, the findings from the current set of experiments are consistent with previous work indicating that corvids struggle to use perceptual-motor feedback to solve problems which do not follow intuitive causal rules, and support our suggestion that NC crows used causal cues to pass those tasks on which they were successful.

There are, however, two explanations for these similar results. The first possibility, which we are not able to rule out here, is that birds are less able to use perceptual-motor feedback when they need to focus their attention on more than one location. For example, in [48], the string which must be pulled is located above the perch, and the reward hangs below. In the U-tube, the tube into which stones can be dropped is separate from the tube which contains the reward. A difficulty attending to all the relevant features of the tasks could explain the poor performance of corvids on the U-tube and the counter-intuitive string pulling problems. This could also explain the difference in performance of children and corvids on the U-tube. The U-tube is comparatively much larger for the birds than for the children, and they must use their beaks to manipulate the objects. Whereas children could view all the components of the U-tube simultaneously from a distance, the birds could not; therefore, attending to perceptual-motor feedback in this task could have been specifically more difficult for the birds. Alternatively, corvids may require both intuitive causal information and perceptual-motor feedback to pass a task, and fail when one of these requirements is not met, supported by the series of stone dropping experiments conducted with Eurasian jays [30]. Given that the NC crows failed the U-tube in the current study, we should determine whether birds still fail a version of this task when all the relevant task components are clearly presented within the birds' line of sight. This would provide stronger evidence that it is the counter-intuitive causal information, and not the perceptual layout of the task, which impairs the birds' performance.

There are other factors which could have influenced the crows' performance on the U-tube[49]. Here, the identity of the connected tube was indicated by an arbitrary cue: red or blue markers. Although attending to this cue was unnecessary if birds could rely solely on perceptual-motor feedback, for all other successful strategies subjects had to associate movement or causal cues with the arbitrary colour of the tubes, which might have been easier for the children. Furthermore, the tubes in the U-tube apparatus were not as spatially separated as in other experiments (7 cm apart, as opposed to 30 cm apart in Exps. 1, 4 & 5), which may have made it harder for birds to inhibit unsuccessful actions towards them (see [49]), or to distinguish between the tubes. The children's superior performance could also reflect a fundamental difference in the cognitive abilities of corvids and humans. Many successful children inferred a hidden connection between the two tubes, yet it has been claimed that non-human animals are incapable of making inferences about unobservable causes [50]. Thus, although NC crows' are able to reason about hidden causal agents [41], reasoning about hidden causal mechanisms may be cognitively beyond corvids' grasp. Although, alternatively, the ability to infer the hidden connection could predominantly stem from children's prior experience with hidden causes, or with containers of water, which is likely to greatly exceed the crows'.

We do not yet know if NC crows could pass the U-tube with modifications to address the limitations above. In particular, would their performance improve if we presented birds with spatially separated tubes, brought the locations of the stone drops and the reward closer together, or modified the use of arbitrary cues? Equally, we do not know if they could pass this task if the hidden mechanism was explicitly revealed; i.e. do NC crows understand, in principle, that dropping stones into a connected tube could bring a floating reward within reach? Such experiments could help to pinpoint exactly why birds failed the U-tube, and indicate whether the birds follow a heuristic such as ‘objects raise the water level’ – suggested by their performance on the narrow vs. wide task – or whether they can recognise some of the causal properties underlying the displacement of water in differently shaped tubes.

Overall, the results of our six experiments suggest that NC crows do possess a causal understanding of displacement, but this understanding has limits. The NC crows we tested here could not respond appropriately to functional differences in the volume of tubes, nor could they infer the presence of a hidden connection in the U-tube apparatus. Their ability to select appropriate objects to drop into tubes, however, was very robust. They reliably discriminated between sinking and floating objects, and between solid and hollow objects, selecting the correct option almost 90% of the time on these tasks. They also preferred to drop objects into water rather than sand, and into tubes with high rather than low water levels. Furthermore, their inability to pass the U-tube suggests that these crows may possess a level of causal understanding which prevented them from learning rules involving counter-intuitive causal cues, although this should be confirmed by further research. The ability to detect and respond to relevant causal properties demonstrated here, is striking, in spite of its limits, and rivals that of 5–7 year old children [29].

The Aesop's Fable paradigm has so far been applied successfully to understand cognition in rooks, Eurasian jays, four-ten year old children and NC crows [23], [29]–[31]. However, to date, although chimpanzees and orangutans have used water as a tool to raise water level [25], [26], their understanding of displacement in this similar task has not been tested. This method of studying cognition has strong potential for comparative work as it does not centre on a behaviour that only some species perform in the wild. Nor does it require human involvement or demonstration. Any animal capable of picking up stones could potentially participate, and – once trained to drop stones – few trials are required to assess performance. Given that it is unlikely that any animals drop stones to raise water levels in the wild, this paradigm can assess to what extent different species demonstrate an understanding of displacement, without the task having more ecological relevance for some species than others. To date both Eurasian jays and NC crows have failed the U-tube, suggesting that there is a cognitive limit on the level of causal information processing possible by these species. Whether this limit is common to corvids, common to birds, or indeed perhaps common to all non-human animals, is yet to be discovered. The Aesop's Fable paradigm can help us answer this, and so help establish how the ability to process causal information has evolved across the animal kingdom.

## Supporting Information

Data S1 **A trial-by-trial record of the choices made by each bird, for all six experiments.**
(XLSX)

Figure S1 **Diagrams of the training apparatus.** Two apparatuses were used to train birds to drop stones down tubes. A: a baited platform – held in place with magnets – would collapse when a stone was dropped in the tube. B: a pivotal platform – with hidden counterbalancing weights – would swing downwards when 2–4 stones were dropped down the tube.
(TIF)

Movie S1 **Example trials for each of the six experiments.**
(MP4)
