# Supplementary figures and images for: Using the Aesop's Fable Paradigm to Investigate Causal Understanding of Water Displacement by New Caledonian Crows
Source: PLoS One. 2014 Mar 26;9(3):e92895. doi: 10.1371/journal.pone.0092895 (PMC3966847; doi:10.1371/journal.pone.0092895)

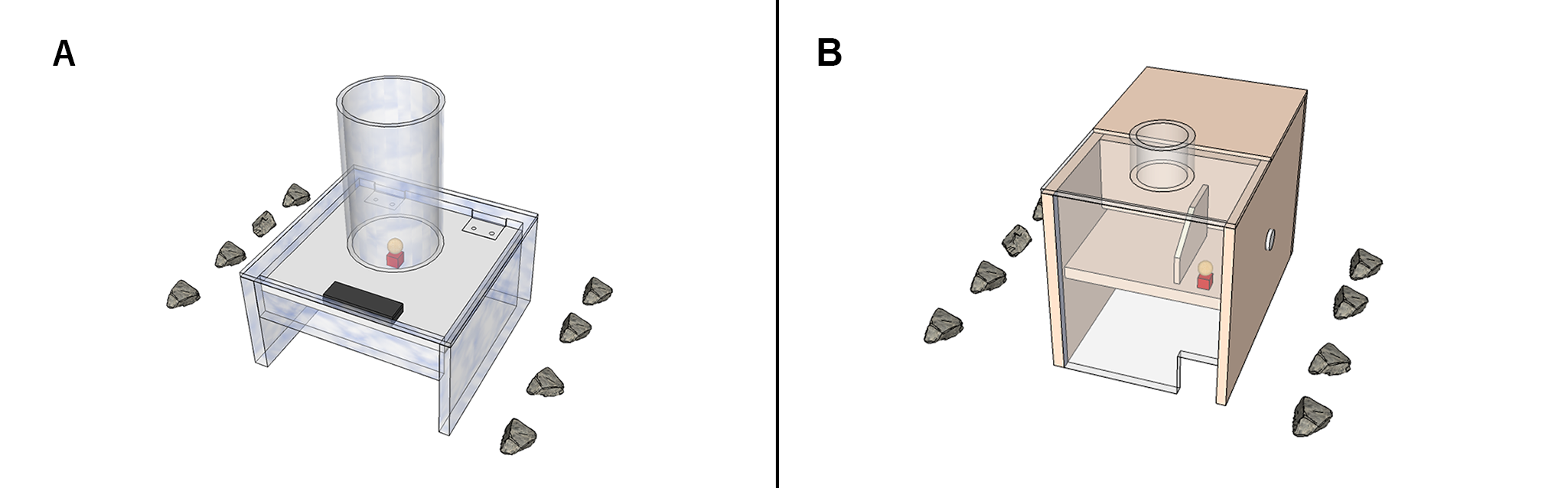

Supplement: Figure S1 — Diagrams of the training apparatus. Two apparatuses were used to train birds to drop stones down tubes. A: a baited platform – held in place with magnets – would collapse when a stone was dropped in the tube. B: a pivotal platform – with hidden counterbalancing weights – would swing downwards when 2–4 stones were dropped down the tube. (TIF) [file pone.0092895.s002.tif]
